# What Matters Most? Developing a Core Patient Reported Outcome Set for Individuals With Genetic Intellectual Disabilities: An International Delphi Study

**DOI:** 10.1111/jir.70081

**Published:** 2026-01-28

**Authors:** Nadia Y. van Silfhout, Maud M. van Muilekom, Leonie A. Menke, Clara D. van Karnebeek, Lotte Haverman, Agnies M. van Eeghen

**Affiliations:** ^1^ Emma Children's Hospital, Department of Child and Adolescent Psychiatry & Psychosocial Care Amsterdam UMC Location University of Amsterdam Amsterdam the Netherlands; ^2^ Emma Children's Hospital, Department of Paediatrics, Amsterdam Gastroenterology Endocrinology Metabolism Amsterdam UMC Location University of Amsterdam Amsterdam the Netherlands; ^3^ Emma Center for Personalized Medicine Amsterdam UMC Amsterdam the Netherlands; ^4^ Mental Health and Personalized Medicine Amsterdam Public Health Research Institute Amsterdam the Netherlands; ^5^ Child Development Amsterdam Reproduction & Development Research Institute Amsterdam the Netherlands; ^6^ Mental Health and Digital Health Amsterdam Public Health Research Institute Amsterdam the Netherlands; ^7^ Aging & Later life and Personalized Medicine Amsterdam Public Health Research Institute Amsterdam the Netherlands; ^8^ Advisium 's Heeren Loo Amersfoort the Netherlands

**Keywords:** Delphi study, intellectual disabilities, patient reported outcome measures, patient reported outcomes, rare genetic neurodevelopmental disorders

## Abstract

**Background:**

Improving care and research for individuals with genetic intellectual disabilities (GID) requires the identification and measurement of relevant patient reported outcomes (PROs). PROs represent the patient perspective on their health status. Currently, a myriad of potentially irrelevant PROs is being measured for individuals with GID. Therefore, the aim of this study is to identify the most relevant PROs through a Delphi survey and consensus meetings and develop a generic core PRO set applicable to the whole GID population to be used in care and research.

**Methods:**

PROs, previously identified through a comprehensive literature review and a qualitative study, were integrated and conceptualised into a pilot generic core PRO set with an expert group. This pilot set was presented in a two‐round Delphi survey with individuals with GID, caregivers and experts. Consensus was set at 60% or more of all participant groups rating a PRO as important for inclusion in the final core PRO set, or as not important for exclusion. The Delphi surveys were followed by two consensus meetings with individuals with GID, caregivers and experts to reach consensus on the undecided PROs.

**Results:**

Twelve individuals with GID, 21 caregivers and 28 experts (total *n* = 61) participated in the first Delphi round. Twenty‐nine PROs were presented to the participants. In the first round, consensus was reached on one important PRO ‘fatigue’. In the second round, consensus was reached on 12 important PROs: fatigue, sleep, physical functioning/activities, quality of life, social functioning/participation, perceived health, cognitive functioning, depressive symptoms, mobility/functioning of the lower extremities, receptive communication, expressive communication and sensory overresponsivity. During the consensus meetings, consensus was reached on seven additional important PROs: pain, anxiety/stress, anger, vision, hearing, gastrointestinal symptoms and mental functioning. This resulted in a final generic core PRO set including the 19 PROs.

**Conclusions:**

This study identified the most relevant PROs for GID, marking the final step in developing a generic core PRO set for the whole GID population. This GID core PRO set provides a framework to guide care, research and policymaking. The next step involves selecting and validating corresponding patient reported outcome measures (PROMs) to adequately measure these PROs: the GID core PROM set.

## 1 Background

Rare genetic neurodevelopmental disorders and/or intellectual disabilities (ID), collectively called genetic intellectual disabilities (GID), affect about 1%–3% of the population (Castrén et al. 2012; Maulik et al. 2011). These conditions, such as fragile X syndrome, tuberous sclerosis complex (TSC), Rett syndrome, and ID without known aetiology, are often characterized by complex and severe physical and neuropsychiatric manifestations such as motor impairments, epilepsy, autism spectrum disorders, and behavioural challenges (Arron et al. 2011; Boot et al. 2015; Lubbers et al. 2022; Robertson et al. 2015). These manifestations can substantially affect daily life, limiting participation in activities and social relationships (Borland et al. 2020; Dawalt et al. 2019).

Care for individuals with GID is complex. Individuals often require intensive, long‐term, and multidisciplinary care, which burdens affected individuals, parents, and other caregivers (Dawson et al. 2016; Fitzgerald and Gallagher 2022). Despite these challenges, personalized care for GID is becoming more available as the awareness of affected individuals' complex needs grows (Huisman et al. 2024). Research involving individuals with GID is challenging as well due to their rarity, great phenotypic variability, and cognitive impairments (Feldman et al. 2014; Mishra and Venkatesh 2024). These factors hinder robust study design, resulting in limited research and contributing to health disparities in this already vulnerable population group. However, technological advances increasingly allow identification of aetiological diagnoses, creating new opportunities for targeted interventions for specific GIDs (van Eeghen et al. 2022). These targeted interventions, such as gene therapy in Rett syndrome and mTOR inhibitors in TSC (Luo et al. 2022; Palmieri et al. 2023), are increasingly being applied and may offer hope for individuals with conditions that were previously untreatable (Henderson et al. 2024).

To guide and improve care and research for individuals with GID, it is important to identify and measure relevant patient reported outcomes (PROs). PROs, such as pain or participation, directly capture the patient's perspective on their health (FDA 2006). By first identifying the most relevant PROs for patients, care, research, and policy can better align with their concerns and needs. For example, if anxiety is identified as a relevant PRO, care pathways can be developed to address it more effectively.

PROs can be measured with patient reported outcome measures (PROMs). PROMs are standardised questionnaires that allow patients to report on various aspects of their health status without interference of a clinician or anyone else (FDA 2006). Incorporating PROMs in care gives clinicians greater insight into individuals' daily functioning and overall well‐being, allowing them to offer personalized treatment and improve overall care strategies (Basch et al. 2013). In addition, incorporating PROMs into research provides comprehensive data on treatment efficacy and disease progression beyond the traditional outcomes (FDA 2006). These PRO data can subsequently inform care and guidelines, shape research agendas and health policy, and support drug approval and treatment reimbursement decisions (Rivera et al. 2019).

Regulatory agencies such as the European Medicines Agency (EMA) and the Food and Drug Administration (FDA), along with patient organizations, increasingly advocate for integrating PRO data into drug development and regulatory processes (Compendium 2022; EMA 2022; FDA 2024). Recent initiatives in the United States and Europe also reflect this priority. For example, the European Rare Disease Research Coordination and Support Action (ERICA) established a repository to harmonize and centralize clinical outcome instruments, including PROMs, for rare diseases (ERICA 2023). Similarly, the Rare Disease Clinical Outcome Assessment Consortium (RD‐COAC) identified scientifically robust instruments, including PROMs, to collect clinically meaningful data in rare disease trials (RD‐COAC 2023). Clinical trials, including those involving individuals with GID, are also incorporating PROMs to assess treatment efficacy from the patients' perspective (Crossnohere et al. 2025; Djukic et al. 2016; Maeder et al. 2022; Müller, Den Hollander, et al. 2024).

The growing focus on PROs amplifies patients' voices and makes outcomes potentially more relevant. However, currently a wide array of PROs is being measured for GID (Müller, van Silfhout, et al. 2024; van Silfhout, van Muilekom, van Karnebeek, Daams, et al. 2025). This reflects the challenge of selecting and measuring the most relevant PROs for individuals with GID, given their phenotypic variability, particularly in cognitive functioning, which can range from profound ID to normal intellectual functioning. Consequently, combining and comparing PRO data becomes difficult, and concerns are raised about whether the most important PROs are being measured. Therefore, there is a need to harmonize PROs for GID and develop a generic core PRO set applicable to the entire GID population. This is particularly important given the impossibility of developing a core PRO set for more than 1500 GIDs (Maia et al. 2021). And although GID represents a complex and heterogeneous population, they also share common comorbidities that may lead to overlapping PROs. Moreover, increasing evidence suggests that PROs, such as fatigue and depressive symptoms, often overlap across conditions and age groups (Katon and Ciechanowski 2002; Swain 2000), supporting the feasibility of developing a generic core PRO set for the entire GID population.

This core PRO set should eventually be part of a future core outcome set (COS). Such a COS would serve as an agreed, standardised set of outcomes that should be measured and reported in all clinical trials involving individuals with GID (Williamson et al. 2012). Beyond clinical trials, this COS could also be useful in other types of research and in routine care, where it helps healthcare professionals monitor relevant outcomes consistently and provide patients with information about their progress (Clarke 2007). The COS for GID will include not only PROs but also other outcomes, such as clinician‐reported outcomes and performance outcomes, and can be adjusted for various conditions or comorbidities.

Previously, we conducted scoping reviews and a qualitative study to identify potentially relevant PROs. Among the identified PROs were perceived health, cognitive functioning, anxiety, and depressive symptoms (van Silfhout, van Muilekom, van Karnebeek, Daams, et al. 2025; van Silfhout, van Muilekom, van Karnebeek, Haverman, and van Eeghen 2025). This study aims to identify the most relevant PROs from the perspectives of affected individuals, caregivers, and experts, representing the final step towards developing a generic core PRO set for children and adults with GID to be used in care and research (van Silfhout et al. 2024).

## 2 Methods

**FIGURE 1:**
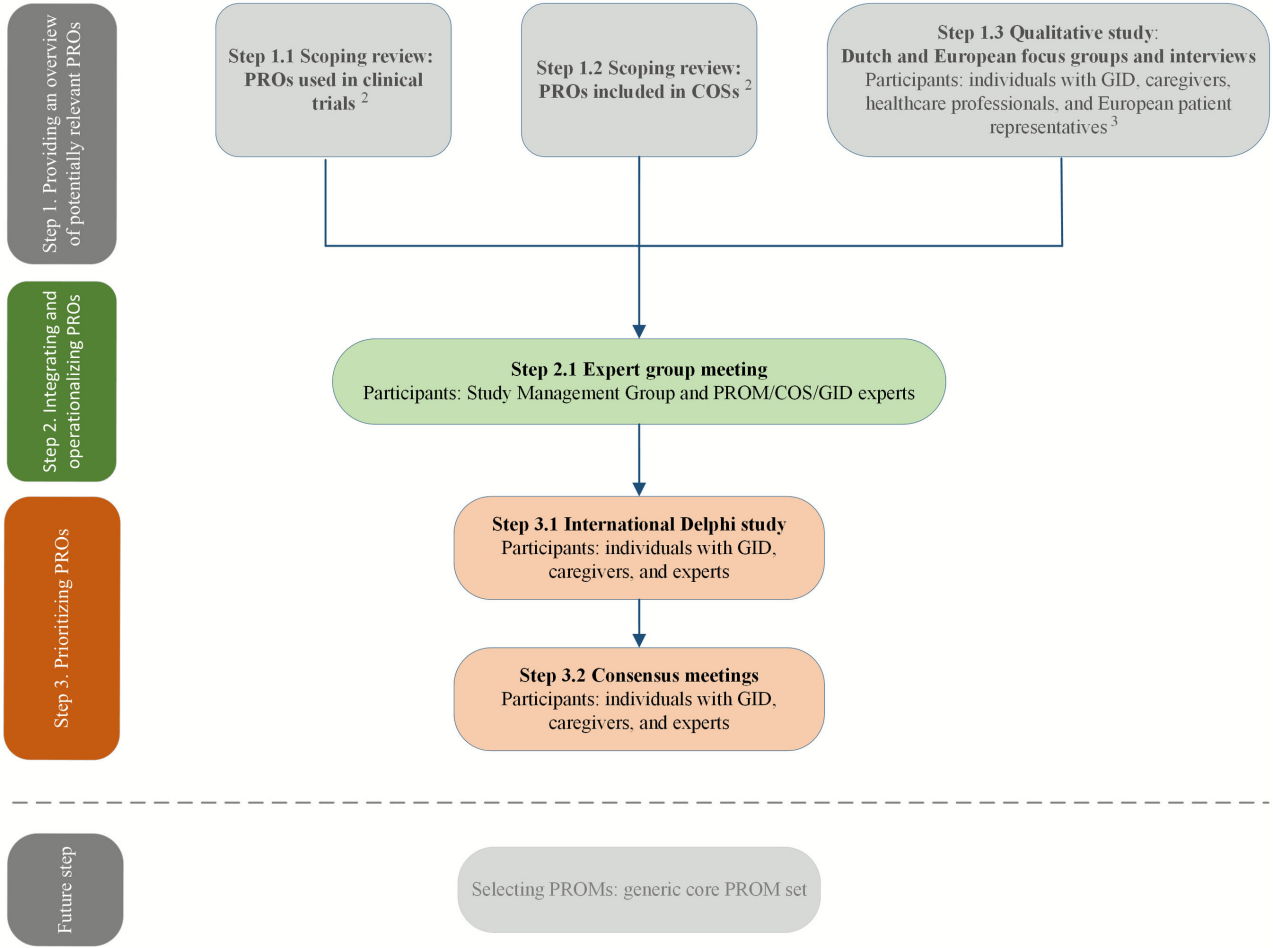
Steps for developing the generic core PRO set: Steps 2.1, 3.1 and 3.2 were undertaken in this study. PROs, patient reported outcomes; PROM(s), patient reported outcome measure(s); COS(s), core outcome set(s); GID, genetic intellectual disabilities. Reproduced/adapted from van Silfhout et al. (2024) under the Creative Commons licence (https://creativecommons.org/licenses/by/4.0/). ^2^Müller, van Silfhout, et al. (2024) and van Silfhout, van Muilekom, van Karnebeek, Daams, et al. (2025). ^3^van Silfhout, van Muilekom, van Karnebeek, Haverman, and van Eeghen (2025).

We performed an expert group meeting followed by a two‐round international Delphi study and two consensus meetings to reach consensus on the most important PROs for individuals with GID (see Figure 1). This study followed the standards for developing and reporting a COS (Kirkham et al. 2017, 2016) and was registered at the Core Outcome Measures in Effectiveness Trails (COMET) Initiative website (https://www.comet‐initiative.org/Studies/Details/3148). A formal review and waiver was obtained for this study by the Medical Ethics Committee of the Amsterdam UMC (W22_345 # 22.413). Participants in the Delphi study and the consensus meetings provided written (electronic) consent before participation.

### 2.1 Providing an Overview of Potentially Relevant PROs

Following the steps outlined in our protocol (van Silfhout et al. 2024), PROs, including their conceptualisation (i.e., content of each PRO), were identified by scoping reviews and through focus groups and interviews with affected individuals, caregivers and clinical experts (i.e., Dutch healthcare professionals, such as psychologists and ID physicians, and European patient representatives) (van Silfhout, van Muilekom, van Karnebeek, Daams, et al. 2025; van Silfhout, van Muilekom, van Karnebeek, Haverman, and van Eeghen 2025). PROs were integrated by two members of the research team (NvS, MvM) and classified and conceptualised within a conceptual framework. This conceptual framework was described and illustrated in our protocol (van Silfhout et al. 2024) and is based on the model of Valderas and Alonso (2008) (combination of the classification model of Wilson and Cleary (1995) and the International Classification of Functioning, Disability and Health (ICF) (Ustun et al. 2003)), and the Patient‐Reported Outcomes Measurement Information System (PROMIS) (Cella et al. 2007). The decision to combine the Valderas and Alonso model, which focuses on PROs, with the PROMIS framework, which focuses on PROMs, was made by experts in the field of PROs, PROMs and PROM development, together with representatives of umbrella organizations, including medical specialists, psychologists, patients and researchers (Oude Voshaar et al. 2023). In this framework, a PRO domain represents a broad PRO category (e.g., mental functioning), while a PRO subdomain represents a specific PRO within a PRO domain (e.g., depressive symptoms). The conceptualisation of each PRO was further supplemented using information from existing literature regarding GID and clinical guidelines for GID. In cases where there was no information about the conceptualisation of a PRO for GID, the general conceptualisation for a PRO used by PROMIS was applied.

### 2.2 Expert Group Meeting

An expert group meeting was performed with the members of the research team (NvS, PhD candidate and psychologist; AvE, ID physician; and MvM, PROM expert and psychologist) and PROM/COS/GID experts from the Amsterdam UMC to discuss the potentially relevant PROs for GID. The research team invited these experts due to their comprehensive knowledge on PROMs, COS, or GID. The classification and conceptualisation of each PRO was discussed during the expert group meeting. If needed, PROs were eliminated (e.g., duplicate or no PRO), and the classification or conceptualisation of the PROs was adjusted. When uncertainty remained during the expert group meeting regarding the classification or conceptualisation of specific PROs, these cases were discussed with another member of the research team (LH, PROM expert and psychologist) and a wider research team with expertise in PROMs. This resulted in a clear and well‐defined set of PROs: the pilot generic core PRO set and a list of excluded PROs or other outcomes. Each PRO domain and PRO subdomain was counted as an individual PRO.

### 2.3 International Delphi Study

#### 2.3.1 Participants

Adolescents (12 years or older) and adults with GID, caregivers, and experts (i.e., international healthcare professionals and patient representatives) could participate in the Delphi study. Affected individuals eligible for inclusion had to have a diagnosis of GID or ID without known aetiology. Because GID includes individuals with rare genetic neurodevelopmental disorders, some may have normal intellectual functioning. These participants were still eligible to take part in the study. Additionally, affected individuals were required to be proficient in reading and writing, as well as capable of reflecting on and providing thoughtful responses to the PROs. Caregivers were eligible if they provided care and support to an individual diagnosed with GID. Experts were eligible if they had at least 5 years' experience working with individuals with GID or working for a patient organization dedicated to a specific GID subgroup and were required to be proficient in English. Participants of the recently performed focus groups and interviews were contacted again if they had given their consent. In addition, affected individuals and caregivers were recruited through healthcare organizations (e.g., Amsterdam UMC and 's Heeren Loo), social media and patient organizations. Experts were recruited through healthcare organizations (e.g., Amsterdam UMC and 's Heeren Loo), social media and the European Reference Network on Intellectual Disability, TeleHealth, Autism and Congenital Anomalies (ERN‐ITHACA), which is a patient‐centred network focusing on specialised and multidisciplinary care for individuals with rare malformation syndromes and rare intellectual and other neurodevelopmental disorders. Interested individuals, caregivers and experts were provided with an information letter outlining the purpose of the study (i.e., reaching consensus on the most relevant PROs). Although the study invitation specified the eligibility criteria, the research team also verified eligibility after application using participants' sociodemographic information.

#### 2.3.2 Survey

The pilot generic core PRO set, including the conceptualisation of each PRO, was presented in the Delphi survey. The Delphi survey for individuals with GID and caregivers was provided in Dutch, whereas the survey for experts was provided in English. The survey for individuals with GID was written in easy‐read language, using simple words and short sentences. The survey was pilot‐tested by one member of each participant group (i.e., by an individual with GID, caregiver and expert) to ensure that the survey was clearly understandable and easy to complete within the predefined time span. The survey response options were also tested to ensure they adequately reflected participants' ratings. The following adjustments were made after the pilot test: The conceptualisation of each PRO was displayed immediately after the PRO, a concluding sentence was added at the end of the survey, and the time span of the survey for individuals with GID was adjusted to 1 h instead of the earlier set time span of half an hour. The survey was distributed to the participants through an online web application (Castor EDC). The Delphi rounds took place between November 2023 and April 2024. Participants were given 2 weeks to complete each Delphi round and received two reminders to complete the Delphi round after 1 week and after 2 weeks.

#### 2.3.3 Delphi Rounds

##### 2.3.3.1 Round One

When opening the first survey, participants were shown an instruction form, which also included a link to the list of the excluded PROs and other outcomes along with the rationale for their exclusion. The first survey consisted of three parts. In the first part, demographic information (e.g., level of intellectual functioning of the affected individual, profession of the expert) of the participants was gathered. In the second part, the pilot generic core PRO set was presented to the participants. Participants were asked to decide whether a PRO should always be discussed during consultation or provided treatment for. This formulation was intended to make the study's aim (reaching consensus on the most relevant PROs) more concrete. The concepts of discussion and treatment were combined, as some PROs may lack available treatments despite their relevance, while others may be important to participants who nonetheless prefer not to discuss them during consultations. Response options were ‘Yes’, ‘Unsure/I do not know’ and ‘No’. These response options were chosen to keep the survey easy to complete for individuals with ID. Participants were encouraged to elaborate on their response in an open text field and could provide feedback on the proposed conceptualisation of the PRO in an open text field. In the third part, participants had the opportunity to suggest additional PROs that they believed had not been considered in the pilot generic core PRO set and were not among previously excluded PROs or other outcomes. If at least two participants suggested the same modification to the conceptualisation of a PRO or suggested the same additional PRO, it was included in the second survey. No PROs were dropped in the first Delphi round to allow participants to reassess the PROs and ensure their inclusion or exclusion during the second Delphi round.

##### 2.3.3.2 Round Two

In the second survey, the pilot generic core PRO set, along with feedback from the first round, was presented to the participants. PROs were arranged in order of importance in the survey, from most important (i.e., highest consensus on importance/lowest consensus on unimportance) to least important (i.e., lowest consensus on importance/highest consensus on unimportance). For each PRO, the percentage of participants who rated a PRO as important in round one was provided for each participant group (i.e., for individuals with GID, caregivers and experts). The reasons provided by the participants in the first round for the perceived importance or unimportance of each PRO were summarised and compiled into a document, which was also shared with the participants via a link in the instruction form. Participants were asked to reconsider their decision on whether a PRO should always be discussed during consultation or provided treatment for. Response options were once again ‘Yes’, ‘Unsure/I do not know’ and ‘No’. In this round, participants were not asked to justify why a PRO should always be discussed or treated, nor to respond to its conceptualisation or suggest additional PROs.

#### 2.3.4 Analyses

The results of the Delphi surveys were analysed for each participant group in Microsoft Excel. Given the considerable diversity and variability within and between genetic subgroups, we anticipated a wide range of relevant PROs for this population group. For this reason, the consensus threshold was set lower, namely at 60% or more of all participant groups responding ‘Yes’ to include a PRO in the final generic core PRO set, compared with the 67%–70% threshold often used in other studies (Harman et al. 2015; Mokkink et al. 2020; Prinsen et al. 2018). If 60% or more of all participant groups responded ‘No’ for a PRO, it was not included in the final generic core PRO set. When consensus had not been reached on a PRO, it was discussed during the consensus meetings.

### 2.4 Consensus Meetings

Two consensus meetings were organised in May 2024 to reach consensus on the remaining PROs: one online consensus meeting with individuals with GID and caregivers conducted in Dutch, and one online consensus meeting with experts (i.e., international healthcare professionals and patient representatives) conducted in English. The two consensus meetings were conducted separately to ensure that the perspectives of individuals and caregivers were sufficiently heard without being overshadowed by those of experts. At the end of Delphi round two, participants were asked if they wanted to participate in an online consensus meeting. If too few participants indicated that they wished to participate in the online consensus meeting (i.e., less than three individuals of one of the participant groups), additional individuals with GID, caregivers, and experts were recruited via healthcare or patient organizations, as participation in the Delphi study is not a requirement for attending a consensus meeting (Williamson et al. 2017). During the consensus meetings, the remaining PROs (i.e., on which consensus had not yet been reached after Delphi round two) were shown again to participants in order of importance in a PowerPoint presentation, from most important (i.e., highest consensus on importance/lowest consensus on unimportance) to least important (i.e., lowest consensus on importance/highest consensus on unimportance). For each PRO, the conceptualisation and the percentage of participants rating it as important in Delphi round two were shown for each participant group. Example PROM items were provided to help participants better understand the meaning and questions related to each PRO. PROs were discussed one by one among participants. Then, participants were asked to decide whether the PRO must always be discussed during consultation or provided treatment for. Participants could anonymously make their decision via an online rating tool (Wooclap). Response options were ‘Yes’ or ‘No’. If at least 60% of all participants responded ‘Yes’ for a PRO in both consensus meetings, it was included in the final generic core PRO set. If not, the PRO was excluded.

## 3 Results

### 3.1 Expert Group Meeting

Three members of the research team (NvS, MvM, AvE), one GID expert (LM) and two PROM/COS experts (HvO, CT) participated in the expert group meeting. Several outcomes were eliminated as they constituted no PROs (e.g., ‘dental problems’ and ‘epilepsy’). The conceptualisation of several PROs was adapted (e.g., in the conceptualisation of ‘relationships’, ‘love relationships’ was adapted to ‘love relationships at an older age’, as young children typically do not have romantic relationships) or supplemented (e.g., ‘feeling understimulated or bored due to little stimulation from the environment’ was added to the conceptualisation of ‘sensory underresponsivity’). After discussing all PROs, 29 unique PROs remained; the pilot generic core PRO set (Data S1).

### 3.2 International Delphi Study

#### 3.2.1 Participants

In round one, there were 61 participants, including 12 individuals with GID, 21 caregivers, and 28 experts. In round two, there were 46 participants, including 10 individuals with GID, 14 caregivers and 22 experts. Only participants who responded in round one were invited for round two. Table 1 shows the characteristics of the participants per round.

**TABLE 1:** Characteristics Delphi study participants.

	Round 1	Round 2
Number of participants	61	46
**Individuals with GID** (frequency)	12	10
	Mean (range)	Mean (range)
Age	31.4 (14–55)	28.3 (14–46)
	Frequency	Frequency
Gender (female)^a^	7	6
Diagnosis unknown	3	2
Diagnosis (GID)^b^		
16p11.2 deletion syndrome	2	2
Fragile X syndrome	1	1
Tuberous sclerosis complex	1	1
22.q11.2 deletion syndrome	1	1
Chung–Jansen syndrome	1	1
Joubert syndrome	1	1
Primrose syndrome	1	1
Other^c^	1	0
Intellectual functioning^b^		
Normal	2	2
Borderline	1	1
Mild ID^d^	8	6
Moderate ID^d^	1	1
**Caregivers** (frequency)	21	14
	Mean (range)	Mean (range)
Age child	16.3 (5–38)	14.8 (5–38)
	Frequency	Frequency
Gender child (male)^a^	14	8
Diagnosis unknown	4	3
Diagnosis (GID)^b^		
DeSanto–Shinawi syndrome	1	1
FOXP1 syndrome	1	1
15q13.3 microdeletion syndrome	1	1
Snijders Blok–Campeau syndrome	1	1
Ehlers–Danlos syndrome	1	1
SPTAN1 syndrome	1	1
Coffin–Siris syndrome	1	1
Wiedemann–Steiner syndrome	1	1
Mosaic trisomy 9	1	1
CLTC‐related disorder	1	1
SLC6A1‐related disorder	1	1
Other^c^	6	0
Intellectual functioning^b^		
Normal	0	0
Borderline	1	1
Mild ID^d^	6	4
Moderate ID^d^	6	5
Severe ID^d^	7	4
Profound ID^d^	1	0
**Experts** ^e^ (frequency)	28	22
Profession		
Patient representative	13	9
ID physician	3	3
Psychologist	3	3
Clinical geneticist	3	2
Paediatrician	3	2
Other	3	3
Country		
The Netherlands	8	8
France	4	2
Italy	3	2
United Kingdom	2	2
Romania	2	2
United States	2	1
Other^f^	7	5

Abbreviations: GID, genetic intellectual disabilities; ID, intellectual disabilities.

^a^ None of the individuals with GID identified as a gender outside the binary categories of male or female.

^b^ As reported by affected individuals or caregivers.

^c^ Other diagnosis (each with only one participant in Round 1): Peters plus syndrome, Cornelia de Lange syndrome, fragile X syndrome, Pitt–Hopkins syndrome, 22.q11.2 deletion syndrome, Cowden syndrome and Tatton–Brown–Rahman syndrome.

^d^ Diagnostic and Statistical Manual of Mental Disorders, Fifth Edition (DSM‐5) (American Psychiatric Association and Association 2013).

^e^ Two experts from the expert group meeting also took part in the Delphi study as a clinician (AvE, LM), and one of them (AvE) participated in the expert consensus meeting as a clinician.

^f^ Other country: Hungary, Slovenia, Germany, Norway, Portugal, Belgium, Spain.

#### 3.2.2 Delphi Rounds

In round one, consensus was reached on one out of 29 (3%) PROs considered important (i.e., 60% or more of all participant groups responding ‘Yes’ for a PRO). No consensus was reached on PROs considered not important. The conceptualisation of five PROs (sleep, sensory overresponsivity, sensory underresponsivity, receptive communication, and sexual functioning) was supplemented (e.g., ‘an overresponse to sensory stimuli’ was added to the conceptualisation of ‘sensory overresponsivity’) following responses from two or more experts. No additional PROs were suggested by two or more participants. In round two, consensus was reached on 12 out of 29 (41%) PROs considered important. Again, no consensus was reached on PROs considered not important. As no additional PROs were added to the second survey, a third Delphi round was not conducted. Table 2 shows the percentage of each participant group rating a PRO as important (light green for ≥ 60%) and the consensus reached across all participant groups (dark green for ≥ 60%) in round one and round two. The remaining 17 PROs on which consensus had not been reached were evaluated by two members of the research team (NvS, MvM). The PRO ‘participation/joining’ was eliminated, as the conceptualisation of this PRO overlapped with the conceptualisation of the PRO ‘social functioning/participation’, as noted by some of the participants. This resulted in 16 remaining PROs on which consensus had not yet been reached, and these were discussed during the consensus meetings.

**TABLE 2:** PROs considered important in Delphi round one and two.

	Round 1				Round 2			
PRO	Individuals with GID	Caregivers	Experts	Consensus	Individuals with GID	Caregivers	Experts	Consensus
1. Fatigue	75%	67%	86%	≥ 67%	80%	100%	100%	≥ 80%
2. Sleep	92%	57%	100%	≥ 57%	100%	79%	100%	≥ 79%
3. Physical functioning/activities	50%	71%	96%	≥ 50%	100%	79%	77%	≥ 77%
4. Quality of life	58%	57%	89%	≥ 57%	80%	71%	77%	≥ 71%
5. Social functioning/participation	42%	52%	96%	≥ 42%	70%	79%	82%	≥ 70%
6. Perceived health	92%	52%	75%	≥ 52%	100%	65%	73%	≥ 65%
7. Cognitive functioning	50%	62%	96%	≥ 50%	70%	65%	91%	≥ 65%
8. Depressive symptoms	50%	24%	75%	≥ 24%	70%	64%	86%	≥ 64%
9. Mobility/functioning of the lower extremities	58%	57%	82%	≥ 57%	100%	65%	64%	≥ 64%
10. Receptive communication	50%	62%	82%	≥ 50%	60%	72%	77%	≥ 60%
11. Sensory overresponsivity	50%	86%	75%	≥ 50%	60%	100%	68%	≥ 60%
12. Expressive communication	33%	76%	89%	≥ 33%	60%	64%	68%	≥ 60%
13. Pain	58%	52%	64%	≥ 52%	90%	57%	86%	≥ 57%
14. Anxiety/stress	58%	81%	93%	≥ 58%	80%	57%	100%	≥ 57%
15. Anger/irritability	42%	71%	93%	≥ 42%	50%	93%	86%	≥ 50%
16. Self‐care	33%	76%	93%	≥ 33%	50%	86%	91%	≥ 50%
17. Vision	33%	33%	79%	≥ 33%	70%	50%	82%	≥ 50%
18. Gastrointestinal symptoms	50%	53%	89%	≥ 50%	70%	50%	77%	≥ 50%
19. Respiratory symptoms	25%	38%	71%	≥ 25%	70%	50%	64%	≥ 50%
20. Pain interference	75%	38%	64%	≥ 38%	90%	43%	68%	≥ 43%
21. Functioning of the upper extremities	50%	52%	75%	≥ 50%	80%	43%	59%	≥ 43%
22. Participation/joining	42%	52%	86%	≥ 42%	40%	57%	73%	≥ 40%
23. Relationships	25%	48%	92%	≥ 25%	40%	50%	59%	≥ 40%
24. Chewing and swallowing	25%	43%	64%	≥ 25%	40%	50%	59%	≥ 40%
25. Sensory underresponsivity	25%	48%	61%	≥ 25%	30%	43%	59%	≥ 30%
26. Sexual functioning	25%	33%	79%	≥ 25%	30%	43%	50%	≥ 30%
27. Hearing	25%	24%	71%	≥ 24%	70%	28%	82%	≥ 28%
28. Itch	42%	24%	21%	≥ 21%	40%	36%	27%	≥ 27%
29. Mental functioning	42%	52%	79%	≥ 42%	20%	79%	91%	≥ 20%

*Note:* Light green indicates consensus within a participant group; dark green indicates consensus across all three participant groups.

Abbreviations: GID, genetic intellectual disabilities; PRO, patient reported outcome.

PROs are ranked from highest to lowest consensus on importance in Delphi round two.

### 3.3 Consensus Meetings

The remaining 16 PROs were discussed during the two online consensus meetings. In total, three individuals with GID and three caregivers participated in one consensus meeting, and six experts in the other consensus meeting. One individual with GID and all experts had also participated in the Delphi study. Characteristics of the participants are described in Data S2.

Table 3 shows the percentage of participants in each consensus meeting rating a PRO as important (light green for ≥ 60%) and the final consensus reached across consensus meetings (dark green for ≥ 60%). Consensus was reached on seven additional PROs considered important: pain, hearing, anxiety/stress, gastrointestinal symptoms, anger/irritability, vision, and mental functioning.

**TABLE 3:** PROs considered important in the consensus meetings.

	Consensus meetings		
PRO	Individuals with GID and caregivers	Experts	Consensus
1. Pain	100%	100%	100%
2. Hearing	100%	100%	100%
3. Anxiety/stress	83%	100%	83%
4. Gastrointestinal symptoms	83%	100%	83%
5. Anger/irritability	100%	67%	67%
6. Vision	67%	83%	67%
7. Mental functioning	67%	83%	67%
8. Respiratory symptoms	100%	50%	50%
9. Self‐care	50%	50%	50%
10. Sensory underresponsivity	67%	33%	33%
11. Sexual functioning	33%	33%	33%
12. Relationships	17%	50%	17%
13. Pain interference	100%	0%	0%
14. Chewing and swallowing	67%	0%	0%
15. Itch	50%	0%	0%
16. Functioning of the upper extremities	33%	0%	0%

*Note:* Light green indicates consensus within one consensus meeting; dark green indicates consensus across both consensus meetings.

Abbreviations: PRO, patient reported outcome; GID, genetic intellectual disabilities.

PROs are ranked from highest to lowest consensus on importance.

### 3.4 Final Core PRO Set

The final generic core PRO set included 19 PROs: 12 agreed upon during the Delphi study and seven during the consensus meetings. The classification and conceptualisation of the PROs are presented in Table 4.

**TABLE 4:** Final generic core PRO set.

	PRO domain	PRO domain conceptualisation	PRO subdomain	PRO subdomain conceptualisation
Overarching	Quality of life	Perceived overall quality of life.	None	N.A.
	2 Perceived health	Perceived overall health.	None	N.A.
Functioning	3 Physical functioning/activities	Ability to perform everyday activities.	4 Mobility/functioning of the lower extremities	Activities of physical mobility, such as moving, walking, running, and cycling.
	5 Social functioning/participation	Ability to take part in social roles and activities, being able to engage with others.	None	N.A.
	6 Mental functioning	Overall evaluation of one's mental health.	7 Depressive symptoms	Experienced depressive symptoms, negative mood (e.g., sadness due to feeling overwhelmed), suicidal thoughts.
			8 Anxiety/stress	Experienced symptoms of anxiety, feelings of panic, panic attacks, worry (about the future), tension/stress (due to feeling overwhelmed), nervousness, restlessness, compulsive thoughts, fretting, feeling threatened, fear of being abandoned/being alone, fear of hospital visits and medical procedures, fear of social interaction, fear of not being able to keep up in society/at school, fear of trusting others, insecurity.
			9 Anger/irritability	Experienced feelings of anger, frustration, irritability.
			10 Sensory overresponsivity	An overresponse to sensory stimuli. Avoidance of sensory stimuli. Sensitive to crowded environments, loud noise, lots of noise/language/questions, bright light, strong smell or taste and different textures of food and clothing.
	11 Cognitive functioning	Paying attention/concentrating, quickly processing information, being flexible (accepting changing situations), remembering things. Cognitive decline/loss of cognitive abilities.	None	N.A.
	Communication^a^	N.A.; expressive and receptive communication fully encompass the PRO ‘communication’.	12 Expressive communication	Turning thoughts into words, stuttering, nonverbal communication (facial expressions, gestures, posture).
			13 Receptive communication	Understanding verbal communication/language.
Symptoms	14 Fatigue	Degree (intensity) of fatigue, low energy during the day, quickly tired after physical and/or cognitive activities, falling asleep during the day.	None	N.A.
	15 Sleep	Perceived quality of sleep, sleep–wake rhythm, falling asleep and sleeping through the night, being awake during the night, bedwetting, sleepwalking.	None	N.A.
	16 Pain	Degree (intensity) of pain.	None	N.A.
	17 Hearing	Degree of poor hearing.	None	N.A.
	18 Vision	Degree of poor vision.	None	N.A.
	19 Gastrointestinal symptoms	Constipation, reflux, abdominal pain, nausea, vomiting, flatulence, burping.	None	N.A.

^a^ Not counted as a unique PRO, as the PRO subdomains fully encompass the PRO domain.

## 4 Discussion

The aim of this study was to identify the most relevant PROs from the perspectives of affected individuals, caregivers, and experts through an international Delphi study and consensus meetings. It marks the final step in developing a generic core PRO set for GID, intended for use in both care and research, as previously outlined in our protocol (van Silfhout et al. 2024). In the first Delphi round, consensus was reached on only one important PRO: fatigue. In the second round, consensus was reached on 12 important PROs: fatigue, sleep, physical functioning/activities, quality of life, social functioning/participation, perceived health, cognitive functioning, depressive symptoms, mobility/functioning of the lower extremities, receptive communication, expressive communication, and sensory overresponsivity. One PRO was eliminated, and the 16 PROs on which consensus had not yet been reached during the Delphi rounds were discussed during the consensus meetings. Consensus was reached on seven additional important PROs: pain, hearing, anxiety/stress, gastrointestinal symptoms, anger/irritability, vision, and mental functioning. The final generic core PRO set included 19 PROs related to all life domains, underscoring the need for a holistic approach in GID care and research.

Affected individuals and caregivers were more selective in their ratings than experts, leading to consensus on only one PRO in round one. Almost all PROs were, however, reported by individuals and caregivers in our qualitative study and no additional PROs were suggested by more than one individual or caregiver (van Silfhout, van Muilekom, van Karnebeek, Haverman, and van Eeghen 2025). We therefore consider it likely that this selectivity reflects that individuals and caregivers focused on their own or their child's situation, whereas experts considered the broader heterogeneous GID population. Moreover, as the consensus threshold required ≥ 60% agreement across all participant groups, individuals with GID and caregivers had the casting vote in the final core PRO set.

### 4.1 Integrating the Core PRO Set With Other Evidence

Mental functioning was broadly represented in the core PRO set, including overall mental functioning, depressive symptoms, anxiety/stress, anger/irritability, and sensory overresponsivity. This aligns with our earlier qualitative study, where mental functioning was frequently highlighted by affected individuals, caregivers, and experts as an important topic to discuss during consultation (van Silfhout, van Muilekom, van Karnebeek, Haverman, and van Eeghen 2025). Implementing the core PRO set in care and research will likely enhance focus on mental functioning, potentially leading to more suitable mental healthcare for individuals with GID. Currently, mental healthcare is often inadequate due to fragmented services and a lack of tailored treatment for this population, leading to undiagnosed and untreated mental health issues (Pouls et al. 2023; Schützwohl et al. 2016).

Various symptoms were also broadly represented in the core PRO set. While symptoms like sleep issues, pain, and gastrointestinal problems in individuals with GID are well researched (Agar et al. 2021; De Knegt et al. 2017; Robertson et al. 2018), fatigue remains underexplored in research studies. This might also suggest a gap in GID care. Previous studies in other health conditions, such as heart failure and autoimmune disease, demonstrated that fatigue can significantly affect daily life, impacting physical and mental health, as well as the ability to perform daily tasks and engage in social activities (Elefante et al. 2020; Schjoedt et al. 2016). This underscores the need to address and understand fatigue in GID care and research, a focus that may be addressed through the implementation of the core PRO set.

An interesting finding was that the core PRO set showed a complete overlap with the Dutch standard set of generic PROs, which is currently implemented in medical care (Oude Voshaar et al. 2023). While our core PRO set is more comprehensive, including specific PROs like gastrointestinal symptoms, key general PROs such as fatigue, depressive symptoms, and pain are all part of the generic Dutch set as well. This finding reinforces the evidence that most PROs, such as fatigue and depressive symptoms, are relevant across various conditions and age groups, including those with GID (Katon and Ciechanowski 2002; Swain 2000).

Finally, behaviour emerged as a key outcome for GID in our qualitative study (van Silfhout, van Muilekom, van Karnebeek, Haverman, and van Eeghen 2025) but was not included in this Delphi study. Behaviour is a multidimensional concept and a manifestation of underlying health issues, such as anger, leading to externalizing behaviour, or depression, resulting in internalizing behaviour. By including these underlying health issues in the final core PRO set, behaviour is (partially) captured. However, further research, for example, as part of a validation study, is warranted to see if we missed certain components of behaviour.

### 4.2 The Core PRO Set in Care and Research

While selecting the core PRO set, we were careful to obtain a balance between comprehensiveness and maintaining future usability (Williamson et al. 2017), as especially caregivers are often already overburdened and resistant to large questionnaires. The core PRO set is relatively extensive and diverse compared with other COS. However, it likely reflects the complex and heterogeneous nature of individuals with GID. It is therefore important that this set will be measured using as few items as possible to minimise burden on respondents. In care, the PROs should be measured, assessed, and discussed during consultations with the healthcare professional, with the goal of adequately monitoring the functioning of affected individuals, screening for relevant problems, and establishing personalized treatments. In research, the set should be measured to ensure relevant outcomes for affected individuals.

### 4.3 Strengths and Limitations

A strength of this study is the inclusion of individuals with GID and caregivers in the Delphi study and in one of the consensus meetings, which is not commonly done during full COS development studies (Gargon et al. 2017). Furthermore, the participation of experts from various countries contributed to a broad perspective, enhancing the generalizability of the core PRO set across different countries.

A limitation of this study is that only individuals with GID who were able to complete an online survey and/or reflect on their own responses and those of the participant groups were included in the Delphi study and consensus meeting. Given the challenging nature of this task, the inclusion of affected individuals with a wide range in intellectual functioning was difficult. Although caregivers and experts also represent individuals with severe to profound ID, the perspectives of these individuals are underrepresented, potentially biasing the PROs towards those more relevant to individuals with higher intellectual functioning. Additionally, the included individuals and caregivers represent only a small fraction of the over 1500 identified GIDs. It is also important to note that the insight into why participants changed their responses in Delphi round two could have provided valuable information. Several PROs, initially rated as not important by at least one group, were considered important after receiving feedback from round one. For example, only 24% of the caregivers rated depressive symptoms as important in round one, increasing to 64% in round two. The specific reasons for this shift remain unclear, as participants were not asked to explain why they changed their response. Another limitation is that two individuals with GID and three caregivers who attended the consensus meeting had not participated in the Delphi study. Although participation in the Delphi study was not a requirement, they did not go through the full process and therefore missed feedback from round one, which may have shaped their perspective differently. An additional limitation is that, in Delphi round two, the PROs were ranked and the percentages of each participant group considering a PRO important were displayed, which might have created a bias by steering participants towards the majority opinion rather than adhering to their own. Finally, because Dutch individuals with GID and caregivers were more critical in rating the PROs, they had greater influence on the final core PRO set. The set may therefore reflect their perspectives more strongly than those of the international experts. When implementing the set outside the Netherlands, further validation to assess its relevance and completeness within the target population may be warranted.

### 4.4 Future Research

In the next step, we will focus on selecting the most suitable PROMs to measure the core PRO set. These PROMs should be short, easy to complete, generic and available as both self‐ and proxy‐report. They should be applicable to children and adults, including individuals with learning disabilities and mild to profound ID, to encompass the whole GID population. However, some PROs may still lack an existing available PROM, necessitating further studies to develop an appropriate PROM for this specific PRO. Once the PROMs are selected and the core PROM set is established, validation in the GID population is essential, for example, by assessing reliability, known‐groups validity and responsiveness of the PROMs for both self‐ and proxy‐reports. Ultimately, the core PROM set should be implemented into GID care and research. Implementation researchers will carry out this process, considering potential barriers such as clinicians not discussing PROM results with the patient and/or caregiver during a consultation (Teela et al. 2021; van Muilekom et al. 2022).

The core PRO(M) set could contribute to a future COS that includes all relevant outcomes for GID. During the COS development, it is crucial to re‐evaluate how each PRO is best measured, whether with a PROM or a different instrument. For example, while hearing can be assessed using a PROM, a hearing test might be more appropriate, allowing for future replacement of the PROM with a more suitable instrument. Additionally, the PROs and other outcomes excluded from this study, such as impact on the family, should be evaluated as well, as they may be crucial for inclusion in the full COS for GID.

## 5 Conclusions

This study represents the final step in establishing a generic core PRO set for individuals with GID. Following two scoping reviews and focus groups and interviews with individuals with GID, caregivers, and healthcare professionals (van Silfhout, van Muilekom, van Karnebeek, Daams, et al. 2025; van Silfhout, van Muilekom, van Karnebeek, Haverman, and van Eeghen 2025), key PROs were identified. These outcomes formed the basis for this Delphi study and subsequent consensus meetings, in which 19 core PROs were selected. Together, this GID core PRO set provides a framework to guide clinical care, research and health policy for GID, ensuring better alignment with the needs and priorities of affected individuals, as well as caregivers and healthcare professionals. The next step is to select appropriate PROMs to effectively measure these PROs, forming the core PROM set. Ultimately, this core PROM set will be validated in the GID population and implemented in both care and research. Using this core PROM set will enable us to incorporate the perspectives of affected individuals and to combine and compare relevant PRO data across different GID groups.

## Funding

This study was funded by the ForWis(h)dom Foundation and 's Heeren Loo. They had no role in the conceptualisation, design, data collection, analysis, decision to publish or preparation of the manuscript.

## Ethics Statement

The Medical Ethics Review Committee of Amsterdam UMC determined that the Medical Research Involving Human Subjects Act (WMO) did not apply to this study and that formal ethical approval was not required (W22_345 #22.413). Written informed consent was obtained from all participants.

## Conflicts of Interest

The authors declare no conflicts of interest.

## Supporting information


**Data S1:** Pilot generic core PRO set.

**Data S2:** Characteristics consensus meeting participants.

**Data S3:** Completed Core Outcome Set‐Standards for Reporting.

**Data S4:** Delphi survey experts—round one.

**Data S5:** Delphi survey experts—round two.

**Data S6:** Explanation rating PROs Delphi round one—experts.

## Data Availability

The data used and/or analysed during the current study are available from the corresponding author on reasonable request.
