# Supplementary material for: What Matters Most? Developing a Core Patient Reported Outcome Set for Individuals With Genetic Intellectual Disabilities: An International Delphi Study
Source: J Intellect Disabil Res. 2026 Jan 28;70(4):403–16. doi: 10.1111/jir.70081 (PMC12950628; doi:10.1111/jir.70081)
Supplement: Supplementary file 2 — Data S2: Characteristics consensus meeting participants. [file JIR-70-403-s004.docx]

**Additional file 2.** Characteristics consensus meeting participants

| **Individuals with GID** | N = 3 |
| --- | --- |
|  | Mean (range) |
| Age | 32 (17 – 44) |
|  | Frequency |
| Gender (female) *^1^* | 2 |
| Diagnosis (GID) *^2^* | 2 |
| 16p11.2 deletion syndrome | 1 |
| Fragile X syndrome | 1 |
| Intellectual functioning |  |
| Borderline | 1 |
| Mild ID*^3^* | 2 |
| **Caregivers** | N = 3 |
|  | Mean (range) |
| Age child | 9 (3 – 16) |
|  | Frequency |
| Gender child (male) *^1^* | 2 |
| Diagnosis (GID) *^2^* | 3 |
| Noonan syndrome | 1 |
| Rett syndrome | 1 |
| Smith-Magenis syndrome | 1 |
| Intellectual functioning |  |
| Mild ID*^3^* | 1 |
| Moderate ID*^3^* | 1 |
| Unknown | 1 |
| **Experts** | N = 6 |
| Profession |  |
| Patient representative | 3 |
| ID physician | 1 |
| Psychologist | 1 |
| GID methodologist | 1 |
| Country |  |
| The Netherlands | 2 |
| United States | 1 |
| Hungary | 1 |
| United Kingdom | 1 |
| Slovenia | 1 |

*ID, intellectual disabilities; GID, genetic intellectual disabilities*

*^1^None of the individuals with GID identified as a gender outside the binary categories of male or female*

*^2^As reported by affected individuals or caregivers*

*^3^Diagnostic and Statistical Manual of Mental Disorders, Fifth Edition (DSM-5)*
