# Supplementary material for: What Matters Most? Developing a Core Patient Reported Outcome Set for Individuals With Genetic Intellectual Disabilities: An International Delphi Study
Source: J Intellect Disabil Res. 2026 Jan 28;70(4):403–16. doi: 10.1111/jir.70081 (PMC12950628; doi:10.1111/jir.70081)
Supplement: Supplementary file 3 — Data S3: Completed Core Outcome Set‐Standards for Reporting. [file JIR-70-403-s002.docx]

**Additional file 3.** Completed Core Outcome Set-Standards for Reporting

| **SECTION/TOPIC** | **ITEM NO.** | **CHECKLIST ITEM** | **WHERE REPORTED; PAGE NO.** |
| --- | --- | --- | --- |
| **TITLE/ABSTRACT** |  |  |  |
| Title | 1a | Identify in the title that the paper reports the development of a COS. | Title Page |
| Abstract | 1b | Provide a structures summary. | 1, 2 |
| **INTRODUCTION** |  |  |  |
| Background and Objectives | 2a | Describe the background and explain the rationale for developing the COS. | 2, 3, 4 |
|  | 2b | Describe the specific objectives with reference to developing a COS. | 4, 5 |
| Scope | 3a | Describe the health condition(s) and population(s) covered by the COS. | 5 |
|  | 3b | Describe the intervention(s) covered by the COS. | N.A. |
|  | 3c | Describe the setting(s) in which the COS is to be applied. | 5 |
| **METHODS** |  |  |  |
| Protocol/Registry Entry | 4 | Indicate where the COS development protocol can be accessed, if available, and/or the study registration details. | 5, 6 |
| Participants | 5 | Describe the rationale for stakeholder groups involved in the COS development process, eligibility criteria for participants from each group, and a description of how the individuals involved were identified. | 7, 8 |
| Information Sources | 6a | Describe the information sources used to identify an initial list of outcomes. | 6 |
|  | 6b | Describe how outcomes were dropped/combined, with reasons (if applicable). | 9, 10 |
| Consensus process | 7 | Describe how the consensus process was undertaken. | 9, 10 |
| Outcome scoring | 8 | Describe how outcomes were scored and how scores were summarized. | 9, 10, 11 |
| Consensus definition | 9a | Describe the consensus definition. | 11, 12 |
|  | 9b | Describe the procedure for determining how outcomes were included or excluded from consideration during the consensus process. | 11, 12 |
| Ethics and Consent | 10 | Provide a statement regarding the ethics and consent issues for the study. | 5, 6 |
| **RESULTS** |  |  |  |
| Protocol Deviations | 11 | Describe any changes from the protocol (if applicable), with reasons, and describe what impact these changes have on the results. | N.A. |
| Participants | 12 | Present data on the number and relevant characteristics of the people involved at all stages of COS development. | 13, 14 |
| Outcome | 13a | List all outcomes considered at the start of the consensus process. | 13 |
|  | 13b | List the outcomes in the final COS. | 15, 16 |
| COS | 14 |  |  |
| **DISCUSSION** |  |  |  |
| Limitations | 15 | Discuss any limitations in the COS development process. | 19, 20 |
| Conclusions | 16 | Provide an interpretation of the final COS in the context of other evidence, and implications for future research. | 17, 18, 20, 21 |
| **OTHER INFORMATION** |  |  |  |
| Funding | 17 | Describe sources of funding/role of funders. | 22 |
| Conflict of Interest | 18 | Describe any conflict of interest within the study team and how these were managed. | N.A. |
