# Supplementary material for: What Matters Most? Developing a Core Patient Reported Outcome Set for Individuals With Genetic Intellectual Disabilities: An International Delphi Study
Source: J Intellect Disabil Res. 2026 Jan 28;70(4):403–16. doi: 10.1111/jir.70081 (PMC12950628; doi:10.1111/jir.70081)
Supplement: Supplementary file 4 — Data S4: Delphi survey experts—round one. [file JIR-70-403-s003.docx]

**Additional file 4.** Delphi survey experts – round one

**Instruction**

In this Delphi survey we use the following definition:

- Patient reported outcomes (PROs) are aspects of a patient’s health status, such as symptoms, physical/mental/social functioning, and quality of life.

The questionnaire consists of three parts:

- In part A we ask some demographic information from you as respondent.
- In part B we present 29 PROs for individuals with rare genetic disorders and/or intellectual disability subdivided into different health domains. We also propose a definition of the PROs. Please indicate whether you think a PRO is important or not, and please further explain your answer. You also have the opportunity to improve the definition of a particular PRO if you think certain information is not included in the definition. You can answer in your own language.
- In part C we give you the opportunity to add one or more PROs you consider as key for individuals with rare genetic disorders and/or intellectual disability as well. Important: the PRO(s) should not be part of the list of other important topics (see link ‘Other important topics’).

Here you can find the list of the other important topics: [Link]

Here you can find additional information about the study: [Link]

We use the database Castor to distribute the questionnaires. You can go forward or backward in the questionnaire simply by pressing the button NEXT or PREVIOUS. You can answer the survey on multiple sessions, the questionnaire will open where you left it whenever you access the link from the *same computer*. Once you have completed the questionnaire, press the button SEND.

We thank you very much for your precious time!

Informed consent

- I read the information letter. I was also able to ask questions. I had enough time to decide whether I wanted to participate.

- I know that participation is voluntary. I also know that I can decide at any time not to participate or to stop the study. I do not have to give a reason.

- I consent to the collection and use of my data in the manner and for the purposes specified in the information letter.

- I want to participate in this study.

Please check yes below:

☐ Yes

Round 1

**Part A: Demographic information**

1. In which country are you working?

………………………………..

2. What is your profession?

☐ Paediatrician

☐ Intellectual disability physician

☐ Clinical geneticist

☐ Psychiatrist

☐ Psychologist / behavioural therapist

☐ Patient representative

☐ Other, namely ………………………………………………………..

3. How many years of work experience do you have in this field?

…………………………………………….

4. What type of organization are you currently employed in?

☐ Hospital (any department including psychiatry)

☐ Psychiatric facility adults

☐ Psychiatric facility children and adolescents

☐ Organization for individuals with intellectual disability

☐ Patient organization

☐ Other, namely ………………………………………………….

5. What genetic syndrome do you have experience with?

☐ 16p11.2 deletion syndrome

☐ 22q11.2 deletion syndrome

☐ Cornelia de Lange syndrome

☐ Down syndrome

☐ Fragile X syndrome

☐ Kleefstra syndrome

☐ Pitt-Hopkins syndrome

☐ PHIP / Chung Jansen syndrome

☐ SATB2-associated syndrome

☐ Tuberous sclerosis complex

☐ Williams syndrome

☐ Smith-Magenis syndrome

☐ Other, namely ……………………………..

**Part B: Important PROs**

**DOMAIN A: Symptoms (mental)**

Please indicate whether you think these PROs are important to discuss during consultation or to provide treatment for.

**1. Anxiety/Stress** (Experienced symptoms of anxiety, feelings of panic, panic attacks, worry (about the future), tension/stress (due to feeling overwhelmed), nervousness, restlessness, compulsive thoughts, fretting, feeling threatened, fear of being abandoned/being alone, fear of hospital visits and medical procedures, fear of social interaction, fear of not being able to keep up in society/at school, fear of trusting others, insecurity)

□ Yes

□ Unsure/I do not know

□ No

Explain why you think this PRO is important or not.

………………………………..

Add extra information to the definition of the PRO (only if you think information is missing).

…………………………………

**2. Depressive symptoms** (Experienced depressive symptoms, negative mood (for example: sadness due to feeling overwhelmed), suicidal thoughts)

□ Yes

□ Unsure/I do not know

□ No

Explain why you think this PRO is important or not.

………………………………..

Add extra information to the definition of the PRO (only if you think information is missing).

…………………………………

**3. Anger/irritability** (Experienced feelings of anger, frustration, irritability)

□ Yes

□ Unsure/I do not know

□ No

Explain why you think this PRO is important or not.

………………………………..

Add extra information to the definition of the PRO (only if you think information is missing).

…………………………………

**4. Sensory over-responsivity** (Sensitive to crowded environments, loud noise, lots of noise/language/questions, bright light, strong smell or taste and different textures of food and clothing).

□ Yes

□ Unsure/I do not know

□ No

Explain why you think this PRO is important or not.

………………………………..

Add extra information to the definition of the PRO (only if you think information is missing).

…………………………………

**5. Sensory under-responsivity** (Likes crowds, loud noise, strong flavour and crunchy food, fascinated by sensory stimuli (colours, textures, sounds), processes stimuli from own body (e.g., pain) or the environment (cold/heat) less well. Feels under-stimulated or bored due to little stimulation from the environment)

□ Yes

□ Unsure/I do not know

□ No

Explain why you think this PRO is important or not.

………………………………..

Add extra information to the definition of the PRO (only if you think information is missing).

…………………………………

**6. Expressive communication** (Turning thoughts into words, stuttering, nonverbal communication (facial expressions, gestures, posture))

□ Yes

□ Unsure/I do not know

□ No

Explain why you think this PRO is important or not.

………………………………..

Add extra information to the definition of the PRO (only if you think information is missing).

…………………………………

**7. Receptive communication** (Understanding language)

□ Yes

□ Unsure/I do not know

□ No

Explain why you think this PRO is important or not.

………………………………..

Add extra information to the definition of the PRO (only if you think information is missing).

…………………………………

**DOMAIN B: Symptoms (physical)**

Please indicate whether you think these PROs are important to discuss during consultation or to offer treatment for.

**8. Sleep** (Perceived quality of sleep, sleep-wake rhythm, falling asleep and sleeping through, being awake during the night, bedwetting, sleepwalking)

□ Yes

□ Unsure/I do not know

□ No

Explain why you think this PRO is important or not.

………………………………..

Add extra information to the definition of the PRO (only if you think information is missing).

…………………………………

**9. Degree of pain** (Degree (intensity) of pain)

□ Yes

□ Unsure/I do not know

□ No

Explain on why you think this PRO is important or not.

………………………………..

Add extra information to the definition of the PRO (only if you think information is missing).

…………………………………

**10. Pain interference** (Consequences of pain on relevant aspects of one’s life. This includes the extent to which pain hinders engagement with social, cognitive, emotional, physical, and recreational activities)

□ Yes

□ Unsure/I do not know

□ No

Explain why you think this PRO is important or not.

………………………………..

Add extra information to the definition of the PRO (only if you think information is missing).

…………………………………

**11. Vision** (Degree of poor vision)

□ Yes

□ Unsure/I do not know

□ No

Explain why you think this PRO is important or not.

………………………………..

Add extra information to the definition of the PRO (only if you think information is missing).

…………………………………

**12. Gastrointestinal symptoms** (Constipation, reflux, abdominal pain, nausea, vomiting, flatulence, burping)

□ Yes

□ Unsure/I do not know

□ No

Explain why you think this PRO is important or not.

………………………………..

Add extra information to the definition of the PRO (only if you think information is missing).

…………………………………

**13. Respiratory symptoms** (Hyperventilation, difficulty breathing, coughing, pauses in breathing during sleep or during the day)

□ Yes

□ Unsure/I do not know

□ No

Explain why you think this PRO is important or not.

………………………………..

Add extra information to the definition of the PRO (only if you think information is missing).

…………………………………

**14. Fatigue** (Degree (intensity) of fatigue, low energy during the day, quickly tired after physical and/or cognitive activities, falling asleep during the day)

□ Yes

□ Unsure/I do not know

□ No

Explain why you think this PRO is important or not.

………………………………..

Add extra information to the definition of the PRO (only if you think information is missing).

…………………………………

**15. Chewing and swallowing** (Ability to swallow and/or chew well, excessive chewing, drooling)

□ Yes

□ Unsure/I do not know

□ No

Explain why you think this PRO is important or not.

………………………………..

Add extra information to the definition of the PRO (only if you think information is missing).

…………………………………

**16. Hearing** (Degree of poor hearing)

□ Yes

□ Unsure/I do not know

□ No

Explain why you think this PRO is important or not.

………………………………..

Add extra information to the definition of the PRO (only if you think information is missing).

…………………………………

**17. Itch** (Degree (intensity) of itch)

□ Yes

□ Unsure/I do not know

□ No

Explain why you think this PRO is important or not.

………………………………..

Add extra information to the definition of the PRO (only if you think information is missing).

…………………………………

**DOMAIN C: Functioning**

Please indicate whether you think these PROs are important to discuss during consultation or to provide treatment for.

**18. Physical functioning/activities** (Ability to perform everyday activities)

□ Yes

□ Unsure/I do not know

□ No

Explain why you think this PRO is important or not.

………………………………..

Add extra information to the definition of the PRO (only if you think information is missing).

…………………………………

**19. Mobility/functioning of the lower extremity** (Activities of physical mobility, such as moving, walking, running, and cycling)

□ Yes

□ Unsure/I do not know

□ No

Explain why you think this PRO is important or not.

………………………………..

Add extra information to the definition of the PRO (only if you think information is missing).

…………………………………

**20. Functioning of the upper extremity** (Activities that require use of the upper extremity including shoulder, arm, and hand activities. Ability to make small/precise movements and grabbing things. For example: writing, buttoning, opening a jar)

□ Yes

□ Unsure/I do not know

□ No

Explain why you think this PRO is important or not.

………………………………..

Add extra information to the definition of the PRO (only if you think information is missing).

…………………………………

**21. Self-care/general daily living activities** (Getting up, dressing, hygienic care (for example: bathe or shower yourself), eating independently, going to the toilet independently, and performing (household) chores)

□ Yes

□ Unsure/I do not know

□ No

Explain why you think this PRO is important or not.

………………………………..

Add extra information to the definition of the PRO (only if you think information is missing).

…………………………………

**22. Social functioning/participation** (Ability to take part in social roles and activities, being able to join in with others)

□ Yes

□ Unsure/I do not know

□ No

Explain why you think this PRO is important or not.

………………………………..

Add extra information to the definition of the PRO (only if you think information is missing).

…………………………………

**23. Relationships** (Establishing and maintaining relationships (friendships, family relationships, and at an older age love relationships). Contentment with relationships. Understanding others and their emotions, empathize with others)

□ Yes

□ Unsure/I do not know

□ No

Explain why you think this PRO is important or not.

………………………………..

Add extra information to the definition of the PRO (only if you think information is missing).

…………………………………

**24. Participation/joining** (Participating in society, joining others, joining sport, joining games. Contentment with participation in social activities)

□ Yes

□ Unsure/I do not know

□ No

Explain why you think this PRO is important or not.

………………………………..

Add extra information to the definition of the PRO (only if you think information is missing).

…………………………………

**25. Mental functioning** (Overall evaluation of one’s mental health)

□ Yes

□ Unsure/I do not know

□ No

Explain why you think this PRO is important or not.

………………………………..

Add extra information to the definition of the PRO (only if you think information is missing).

…………………………………

**26. Cognitive functioning** (Paying attention/concentrating, quickly processing information, being flexible (accepting changing situations), remembering things. Cognitive decline/loss of cognitive abilities)

□ Yes

□ Unsure/I do not know

□ No

Explain why you think this PRO is important or not.

………………………………..

Add extra information to the definition of the PRO (only if you think information is missing).

…………………………………

**27. Sexual functioning** (Sexual interest (for example: reduced/no interest or excessive interest), fetishes, satisfaction with sexual functioning (for example: being able to have an orgasm). Sexual inappropriate behaviour (for example: masturbating in inappropriate situations))

□ Yes

□ Unsure/I do not know

□ No

Explain why you think this PRO is important or not.

………………………………..

Add extra information to the definition of the PRO (only if you think information is missing).

…………………………………

**DOMAIN C: Overarching**

Please indicate whether you think these PROs are important to discuss during consultation or to provide treatment for.

**28. Quality of life** (Perceived overall quality of life)

□ Yes

□ Unsure/I do not know

□ No

Explain why you think this PRO is important or not.

………………………………..

Add extra information to the definition of the PRO (only if you think information is missing).

…………………………………

**29. Perceived health** (Perceived overall health)

□ Yes

□ Unsure/I do not know

□ No

Explain why you think this PRO is important or not.

………………………………..

Add extra information to the definition of the PRO (only if you think information is missing).

…………………………………

**Part C: Additional PROs**

The following PRO(s) is/are not among the previous mentioned PROs and not in the list of other important topics, but must be considered as important PRO(s).

…………………………………………….

Thank you so much for completing the survey!

Within a few weeks we will start the second Delphi round. During the second round, will share anonymized and summarized responses from Round 1 and invite you to review and refine your input based on this collective feedback. We look forward to seeing you then!
