# Supplementary material for: What Matters Most? Developing a Core Patient Reported Outcome Set for Individuals With Genetic Intellectual Disabilities: An International Delphi Study
Source: J Intellect Disabil Res. 2026 Jan 28;70(4):403–16. doi: 10.1111/jir.70081 (PMC12950628; doi:10.1111/jir.70081)
Supplement: Supplementary file 5 — Data S5: Delphi survey experts—round two. [file JIR-70-403-s005.docx]

**Additional file 5.** Delphi survey experts – round two

**Instruction**

In this Delphi survey we use the following definition:

Patient reported outcomes (PROs) are aspects of a patient’s health status, such as symptoms, physical/mental/social functioning, and quality of life.

We ask you to reconsider your opinion on the 29 PROs for individuals with rare genetic disorders and/or intellectual disability. For each PRO, the percentage (%) of participants who rated it as important in survey 1 is provided for each stakeholder group (patients, caregivers, and experts). In addition, we arranged the PROs in order of importance in the survey, from most (highest consensus) to least (lowest consensus):

1. Fatigue
2. Anxiety/stress
3. Sleep
4. Quality of life
5. Mobility/functioning of the lower extremities
6. Perceived health
7. Pain
8. Sensory over-responsivity
9. Physical functioning
10. Cognitive functioning
11. Receptive communication
12. Gastrointestinal symptoms
13. Functioning of the upper extremities
14. Anger/irritability
15. Social functioning
16. Participation/joining
17. Mental functioning
18. Pain interference
19. Self-care/general daily living activities
20. Expressive communication
21. Vision
22. Relationships
23. Sensory under-responsivity
24. Chewing and swallowing
25. Respiratory symptoms
26. Sexual functioning
27. Depressive symptoms
28. Hearing
29. Itch

Below, you’ll find the link to the summarized explanations of why participants believed a PRO was important or not important in survey 1.

Link: [Explanations rating PROs survey 1](link) (hold down ctrl+c to open the link in a new tab)

We use the database Castor to distribute the questionnaires. You can go forward or backward in the questionnaire simply by pressing the button NEXT or PREVIOUS. You can answer the survey on multiple sessions, the questionnaire will open where you left it whenever you access the link from the same computer. Once you have completed the questionnaire, press the button SEND.

We thank you very much for your time!

Informed consent

- I read the information letter. I was also able to ask questions. I had enough time to decide whether I wanted to participate.

- I know that participation is voluntary. I also know that I can decide at any time not to participate or to stop the study. I do not have to give a reason.

- I consent to the collection and use of my data in the manner and for the purposes specified in the information letter.

- I want to participate in this study.

Please check yes below:

☐ Yes

**Survey 2: Important PROs**

PRO 1: Fatigue

Below, you’ll find the percentage (%) of participants who considered *fatigue* as an important PRO in survey 1:

Patients: 75%

Caregivers: 67%

Experts: 86%

Please indicate once more whether you think this PRO must always be discussed during consultation or provided treatment for.

**Fatigue** (Degree (intensity) of fatigue, low energy during the day, quickly tired after physical and/or cognitive activities, falling asleep during the day)

□ Yes

□ Unsure/I do not know

□ No

PRO 2: Anxiety/stress

Below, you’ll find the percentage (%) of participants who considered *anxiety/stress* as an important PRO in survey 1:

Patients: 58%

Caregivers: 81%

Experts: 93%

Please indicate once more whether you think this PRO must always be discussed during consultation or provided treatment for.

**Anxiety/Stress** (Experienced symptoms of anxiety, feelings of panic, panic attacks, worry (about the future), tension/stress (due to feeling overwhelmed), nervousness, restlessness, compulsive thoughts, fretting, feeling threatened, fear of being abandoned/being alone, fear of hospital visits and medical procedures, fear of social interaction, fear of not being able to keep up in society/at school, fear of trusting others, insecurity)

□ Yes

□ Unsure/I do not know

□ No

PRO 3: Sleep

Below, you’ll find the percentage (%) of participants who considered *sleep* as an important PRO in survey 1:

Patients: 92%

Caregivers: 57%

Experts: 100%

Please indicate once more whether you think this PRO must always be discussed during consultation or provided treatment for.

**Sleep** (Perceived quality of sleep, sleep-wake rhythm, falling asleep and sleeping through the night, being awake during the night, bedwetting, sleepwalking)

□ Yes

□ Unsure/I do not know

□ No

PRO 4: Quality of life

Below, you’ll find the percentage (%) of participants who considered *quality of life* as an important PRO in survey 1:

Patients: 58%

Caregivers: 57%

Experts: 89%

Please indicate once more whether you think this PRO must always be discussed during consultation or provided treatment for.

**Quality of life** (Perceived overall quality of life)

□ Yes

□ Unsure/I do not know

□ No

PRO 5: Mobility/functioning of the lower extremities

Below, you’ll find the percentage (%) of participants who considered *mobility/functioning of the lower extremities* as an important PRO in survey 1:

Patients: 58%

Caregivers: 57%

Experts: 82%

Please indicate once more whether you think this PRO must always be discussed during consultation or provided treatment for.

**Mobility/functioning of the lower extremities** (Activities of physical mobility, such as moving, walking, running, and cycling)

□ Yes

□ Unsure/I do not know

□ No

PRO 6: Perceived health

Below, you’ll find the percentage (%) of participants who considered *perceived health* as an important PRO in survey 1:

Patients: 92%

Caregivers: 52%

Experts: 75%

Please indicate once more whether you think this PRO must always be discussed during consultation or provided treatment for.

**Perceived health** (Perceived overall health)

□ Yes

□ Unsure/I do not know

□ No

PRO 7: Pain

Below, you’ll find the percentage (%) of participants who considered *pain* as an important PRO in survey 1:

Patients: 58%

Caregivers: 52%

Experts: 64%

Please indicate once more whether you think this PRO must always be discussed during consultation or provided treatment for.

**Pain** (Degree (intensity) of pain)

□ Yes

□ Unsure/I do not know

□ No

PRO 8: Sensory over-responsivity

Below, you’ll find the percentage (%) of participants who considered *sensory over-responsivity* as an important PRO in survey 1:

Patients: 50%

Caregivers: 86%

Experts: 75%

Please indicate once more whether you think this PRO must always be discussed during consultation or provided treatment for.

**Sensory over-responsivity** (An over-response to sensory stimuli. Avoidance of sensory stimuli. Sensitive to crowded environments, loud noise, lots of noise/language/questions, bright light, strong smell or taste and different textures of food and clothing).

□ Yes

□ Unsure/I do not know

□ No

PRO number 9: Physical functioning

Below, you’ll find the percentage (%) of participants who considered *physical functioning* as an important PRO in survey 1:

Patients: 50%

Caregivers: 71%

Experts: 96%

Please indicate once more whether you think this PRO must always be discussed during consultation or provided treatment for.

**Physical functioning** (Ability to perform everyday activities)

□ Yes

□ Unsure/I do not know

□ No

PRO 10: Cognitive functioning

Below, you’ll find the percentage (%) of participants who considered *cognitive functioning* as an important PRO in survey 1:

Patients: 50%

Caregivers: 62%

Experts: 96%

Please indicate once more whether you think this PRO must always be discussed during consultation or provided treatment for.

**Cognitive functioning** (Paying attention/concentrating, quickly processing information, being flexible (accepting changing situations), remembering things. Cognitive decline/loss of cognitive abilities)

□ Yes

□ Unsure/I do not know

□ No

PRO 11: Receptive communication

Below, you’ll find the percentage (%) of participants who considered *receptive communication* as an important PRO in survey 1:

Patients: 50%

Caregivers: 62%

Experts: 82%

Please indicate once more whether you think this PRO must always be discussed during consultation or provided treatment for.

**Receptive communication** (Understanding verbal communication/language)

□ Yes

□ Unsure/I do not know

□ No

PRO 12: Gastrointestinal symptoms

Below, you’ll find the percentage (%) of participants who considered *gastrointestinal symptoms* as an important PRO in survey 1:

Patients: 50%

Caregivers: 53%

Experts: 89%

Please indicate once more whether you think this PRO must always be discussed during consultation or provided treatment for.

**Gastrointestinal symptoms** (Constipation, reflux, abdominal pain, nausea, vomiting, flatulence, burping)

□ Yes

□ Unsure/I do not know

□ No

PRO 13: Functioning of the upper extremities

Below, you’ll find the percentage (%) of participants who considered *functioning of the upper extremities* as an important PRO in survey 1:

Patients: 50%

Caregivers: 52%

Experts: 75%

Please indicate once more whether you think this PRO must always be discussed during consultation or provided treatment for.

**Functioning of the upper extremities** (Activities that require use of the upper extremities including shoulder, arm, and hand activities. Ability to make small/precise movements and grabbing things. For example: writing, buttoning, opening a jar)

□ Yes

□ Unsure/I do not know

□ No

PRO 14: Anger/irritability

Below, you’ll find the percentage (%) of participants who considered *anger/irritability* as an important PRO in survey 1:

Patients: 42%*

Caregivers: 71%*

Experts: 93%*

*NB There is a big difference between patients, caregivers, and experts

Please indicate once more whether you think this PRO must always be discussed during consultation or provided treatment for.

**Anger/irritability** (Experienced feelings of anger, frustration, irritability)

□ Yes

□ Unsure/I do not know

□ No

PRO 15: Social functioning

Below, you’ll find the percentage (%) of participants who considered *social functioning* as an important PRO in survey 1:

Patients: 42%*

Caregivers: 52%*

Experts: 96%*

*NB There is a big difference between patients, caregivers, and experts

Please indicate once more whether you think this PRO must always be discussed during consultation or provided treatment for.

**Social functioning** (Ability to take part in social roles and activities, being able to join in with others)

□ Yes

□ Unsure/I do not know

□ No

PRO 16: Participation/joining

Below, you’ll find the percentage (%) of participants who considered *participation/joining* as an important PRO in survey 1:

Patients: 42%

Caregivers: 52%

Experts: 86%

Please indicate once more whether you think this PRO must always be discussed during consultation or provided treatment for.

**Participation/joining** (Participating in society, joining sport, joining games. Contentment with participation in social activities)

□ Yes

□ Unsure/I do not know

□ No

PRO 17: Mental functioning

Below, you’ll find the percentage (%) of participants who considered *mental functioning* as an important PRO in survey 1:

Patients: 42%

Caregivers: 52%

Experts: 79%

Please indicate once more whether you think this PRO must always be discussed during consultation or provided treatment for.

**Mental functioning** (Overall evaluation of one’s mental health)

□ Yes

□ Unsure/I do not know

□ No

PRO 18: Pain interference

Below, you’ll find the percentage (%) of participants who considered *pain interference* as an important PRO in survey 1:

Patients: 75%

Caregivers: 38%

Experts: 64%

Please indicate once more whether you think this PRO must always be discussed during consultation or provided treatment for.

**Pain interference** (Consequences of pain on relevant aspects of one’s life. This includes the extent to which pain hinders engagement with social, cognitive, emotional, physical, and recreational activities)

□ Yes

□ Unsure/I do not know

□ No

PRO number 19: Self-care/general daily living activities

Below, you’ll find the percentage (%) of participants who considered *self-care/general daily living activities* as an important PRO in survey 1:

Patients: 33%*

Caregivers: 76%*

Experts: 93%*

*NB There is a big difference between patients, caregivers, and experts

Please indicate once more whether you think this PRO must always be discussed during consultation or provided treatment for.

**Self-care/general daily living activities** (Getting up, dressing, hygienic care (for example: bathe or shower yourself), eating independently, going to the toilet independently, and performing (household) chores)

□ Yes

□ Unsure/I do not know

□ No

PRO 20: Expressive communication

Below, you’ll find the percentage (%) of participants who considered *expressive communication* as an important PRO in survey 1:

Patients: 33%*

Caregivers: 76%*

Experts: 89%*

*NB There is a big difference between patients, caregivers, and experts

Please indicate once more whether you think this PRO must always be discussed during consultation or provided treatment for.

**Expressive communication** (Turning thoughts into words, stuttering, nonverbal communication (facial expressions, gestures, posture))

□ Yes

□ Unsure/I do not know

□ No

PRO 21: Vision

Below, you’ll find the percentage (%) of participants who considered *vision* as an important PRO in survey 1:

Patients: 33%

Caregivers: 33%

Experts: 79%

Please indicate once more whether you think this PRO must always be discussed during consultation or provided treatment for.

**Vision** (Degree of poor vision)

□ Yes

□ Unsure/I do not know

□ No

PRO 22: Relationships

Below, you’ll find the percentage (%) of participants who considered *relationships* as an important PRO in survey 1:

Patients: 25%*

Caregivers: 48%*

Experts: 92%*

*NB There is a big difference between patients, caregivers, and experts

Please indicate once more whether you think this PRO must always be discussed during consultation or provided treatment for.

**Relationships** (Establishing and maintaining relationships (friendships, family relationships, and at an older age love relationships). Contentment with relationships. Understanding others and their emotions, empathize with others)

□ Yes

□ Unsure/I do not know

□ No

PRO 23: Sensory under-responsivity

Below, you’ll find the percentage (%) of participants who considered *sensory under-responsivity* as an important PRO in survey 1:

Patients: 25%

Caregivers: 48%

Experts: 61%

Please indicate once more whether you think this PRO must always be discussed during consultation or provided treatment for.

**Sensory under-responsivity** (An under-response to sensory stimuli. Seeking sensory stimuli. Likes crowds, loud noise, strong flavour and crunchy food, fascinated by sensory stimuli (colours, textures, sounds), processes stimuli from own body (e.g., pain) or the environment (cold/heat) less. Feels under-stimulated or bored due to little stimulation from the environment)

□ Yes

□ Unsure/I do not know

□ No

PRO 24: Chewing and swallowing

Below, you’ll find the percentage (%) of participants who considered *chewing and swallowing* as an important PRO in survey 1:

Patients: 25%

Caregivers: 43%

Experts: 64%

Please indicate once more whether you think this PRO must always be discussed during consultation or provided treatment for.

**Chewing and swallowing** (Ability to swallow and/or chew well, excessive chewing, drooling)

□ Yes

□ Unsure/I do not know

□ No

PRO 25: Respiratory symptoms

Below, you’ll find the percentage (%) of participants who considered *respiratory symptoms* as an important PRO in survey 1:

Patients: 24%

Caregivers: 38%

Experts: 71%

Please indicate once more whether you think this PRO must always be discussed during consultation or provided treatment for.

**Respiratory symptoms** (Hyperventilation, difficulty breathing, coughing, pauses in breathing during sleep or during the day)

□ Yes

□ Unsure/I do not know

□ No

PRO 26: Sexual functioning

Below, you’ll find the percentage (%) of participants who considered *sexual functioning* as an important PRO in survey 1:

Patients: 25%*

Caregivers: 33%*

Experts: 79%*

*NB There is a big difference between patients, caregivers, and experts

Please indicate once more whether you think this PRO must always be discussed during consultation or provided treatment for.

**Sexual functioning** (Sexual interest (for example: reduced/no interest or excessive interest), fetishes, satisfaction with sexual functioning, sexual behaviour not matching chronological age)

□ Yes

□ Unsure/I do not know

□ No

PRO 27: Depressive symptoms

Below, you’ll find the percentage (%) of participants who considered *depressive symptoms* as an important PRO in survey 1:

Patients: 50%*

Caregivers: 24%*

Experts: 75%*

*NB There is a big difference between patients, caregivers, and experts

Please indicate once more whether you think this PRO must always be discussed during consultation or provided treatment for.

**Depressive symptoms** (Experienced depressive symptoms, negative mood (for example: sadness due to feeling overwhelmed), suicidal thoughts)

□ Yes

□ Unsure/I do not know

□ No

PRO 28: Hearing

Below, you’ll find the percentage (%) of participants who considered *hearing* as an important PRO in survey 1:

Patients: 25%

Caregivers: 24%

Experts: 71%

Please indicate once more whether you think this PRO must always be discussed during consultation or provided treatment for.

**Hearing** (Degree of poor hearing)

□ Yes

□ Unsure/I do not know

□ No

PRO 29: Itch

Below, you’ll find the percentage (%) of participants who considered *itch* as an important PRO in survey 1:

Patients: 42%

Caregivers: 24%

Experts: 21%

Please indicate once more whether you think this PRO must always be discussed during consultation or provided treatment for.

**Itch** (Degree (intensity) of itch)

□ Yes

□ Unsure/I do not know

□ No

Comments

**Contact information**

Please fill in your first and last name, so we know who completed the survey.

First and last name: ……

Within a few weeks we will organize two consensus meetings to reach consensus on the undecided PROs: one online consensus meeting with experts and one face-to-face consensus meeting with patients and caregivers.

Would you like to participate in the online consensus meeting with experts in April or May? If yes, we will contact you by email.

□ Yes

□ No

Thank you so much for completing the survey!
