# Supplementary material for: What Matters Most? Developing a Core Patient Reported Outcome Set for Individuals With Genetic Intellectual Disabilities: An International Delphi Study
Source: J Intellect Disabil Res. 2026 Jan 28;70(4):403–16. doi: 10.1111/jir.70081 (PMC12950628; doi:10.1111/jir.70081)
Supplement: Supplementary file 6 — Data S6: Explanation rating PROs Delphi round one—experts. [file JIR-70-403-s001.docx]

**Additional file 6.** Explanation rating PROs Delphi round one - experts

**Explanation rating PROs survey 1**

Below, you’ll find the summarized explanations of why participants believed a PRO was important or not important in survey 1 (the responses of patients, caregivers, and experts are combined).

Patient = individual with a rare genetic disorder and/or intellectual disability (ID)

Caregiver = someone who provides care and support to an individual with a rare genetic disorder and/or ID

HCP = healthcare professional of an individual with a rare genetic disorder and/or ID

| **PRO** | **Important** | **Not important** |
| --- | --- | --- |
| 1. **Fatigue** | Fatigue is a very common complaint. Fatigue is often the result of sleeping problems or high melatonin level. Affects sensory processing, (cognitive) functioning and daily activities. Not always discussed by HCPs. Important to assess. | Not a always a big issue. |
| 1. **Anxiety/stress** | Anxiety has a huge impact on health, daily functioning, and quality of life. Anxiety is common among patients. It is an important PRO to discuss with the HCP and finding appropriate treatment to better cope with anxiety. | Not everyone experiences anxiety. Patients have often difficulties with expressing their anxiety. |
| 1. **Sleep** | Sleep problems are very common, especially at a younger age. They affect functioning and quality of life, including that of other family members. Lack of sleep can influence mental well-being (e.g. anger, anxiety), daily activities, and behaviour. Too much sleep can be an indication of depression. Often underreported, undiscussed, underdiagnoses, and underrated. It is important to know when treatment is needed for sleeping problems. | Some patients sleep well. |
| 1. **Quality of life** | Good quality of life is important. Often remains undiscussed with the HCP. Important to consider how the patient can maintain a good quality of life (in the future). | Difficult to define quality of life. Broad PRO. The other PROs describe quality of life. |
| 1. **Mobility/functioning of the lower extremities** | Some patients are not able to walk and are wheelchair-bound. Some patients can only walk with aid. Affects daily functioning and participation. Important to monitor mobility. Mobility can be affected by pain or low muscle tone. | Not always reported by patients or their caregivers. |
| 1. **Perceived health** | Important to measure changes in perceived health, especially before and after treatment. The patient can perceive their health differently compared to how the HCP perceives it. | The other PROs already cover perceived health. Difficult to gain insight into the health of patients from their perspective. |
| 1. **Pain** | Non-verbal patients cannot express their pain adequately, and therefore pain often remains unnoticed. Patients can also have a high or low threshold for pain. Pain is often missed and remains untreated. Pain affects well-being and quality of life. Behaviour problems can be the result of underlying pain. It is important for caregivers to know how they can ease pain. HCPs do not always take pain seriously. | Pain is not that often reported by patients or parents. Pain does not have a relevant impact on patients. |
| 1. **Sensory over-responsivity** | Patients and their caregivers report sensory over-responsivity often. It has an impact on daily functioning. HCPs do not always have much knowledge about this PRO. Sensory over-responsivity is relevant to address during a consultation with the HCP to provide appropriate advice and treatment. | Can a HCP help with this? |
| 1. **Physical functioning** | Problems with physical functioning can affect well-being, social participation, and quality of life. Important aspect for having an independent life. Important to monitor physical functioning to ensure physical problems do not interfere too much with daily life. HCPs do not always address physical functioning during consultation. | Not always relevant. Patients are able to go anywhere with a tricycle bike. |
| 1. **Cognitive functioning** | A huge issue for patients. Affects daily functioning, participation, and well-being. Important to have insight into level of functioning; provide support and adjust the environment as needed. Also provide cognitive challenges in order to allow patients to develop optimally. Important to monitor cognitive functioning in order to identify deterioration. |  |
| 1. **Receptive communication** | Sometimes patients understand much more than they express, or express themselves much better than they understand. Therapy for patients and advice for caregivers may be necessary. Lack of communication can also cause other problems, such as challenging behaviour. Communication is key for social interaction. | Not always an important PRO and sometimes difficult to discuss with someone with ID. |
| 1. **Gastrointestinal symptoms** | Constipation and vomiting is common. Can be a sign of anxiety/stress. Affects behaviour, sleep, well-being, and functioning. Obstipation can also result in pain or shame. | Not all patients and caregivers report gastrointestinal symptoms. Not all patients need a comprehensive gastrointestinal assessment. |
| 1. **Functioning of the upper extremities** | Fine and gross motor skills are often impaired. Affects (cognitive) functioning and participation. Essential to monitor, and, if necessary, to find suitable tools/aid. Important for independence. | Not often seen in older patients. Are there treatment options for this? |
| 1. **Anger/irritability** | Anger can have a huge impact on well-being and functioning, and may lead to isolation. Patients may experience anger, but are not always able to express their anger properly. It is important that the HCP pays attention to anger as well, and provides treatment options. | Not necessary to discuss with a HCP. |
| 1. **Social functioning** | Patients may have difficulties understanding others, and can be afraid of social interaction. Patients often live isolated. They need support in taking part in social life. Social isolation has a high impact on well-being and quality of life. Support from a psychologist may be needed to help patients learn social skills. Gives insight into the level of functioning/ID. Patients can also be too social. | Patients do not always need social contact. Patients can be very social. |
| 1. **Participation/joining** | Patients often have difficulties participating in society, which has a high impact on well-being and quality of life. Support is essential to help patients participate and prevent isolation. Gives insight into the level of functioning/ID. Important to discuss alternative options for participation (e.g. special sports club), and what to expect in the future regarding the participation of patients. | Participation will remain difficult, since patients often don’t like crowds. Can a HCP support with participation? Caregivers often have enough expertise on this PRO. |
| 1. **Mental functioning** | Mental health is very important, since anxiety and depression are common among patients. Behavioural problems can be a sign of underlying mental problems. Mental health affects social interaction, ability to learn, well-being, and quality of life. Psychological support is essential to obtain good mental health. Parents do not always have insight into their child’s internal mental experiences. | Not always a problem. Patients cannot always express their mental issues. |
| 1. **Pain interference** | Pain impacts daily functioning and quality of life. Important to know whether pain hinders engagement in social, cognitive, emotional, physical, and recreational activities. | Not always an issue. |
| 1. **Self-care/general daily living activities** | Patients often need supervision and help with self-care. Self-care must be monitored throughout life; gives insight into daily functioning. Can have a huge impact on well-being and functioning. Important to find assistance with self-care if needed, also to unburden parents/caregivers. Gives insight into the level of functioning/ID. | Does this always need to be discussed with a HCP? |
| 1. **Expressive communication** | Expressive communication is essential for being understood by others. Appropriate therapies should be addressed. Expressive communication problems may lead to problems with social interaction and challenging behaviour. | Not always important and sometimes difficult to discuss with someone with ID. |
| 1. **Vision** | Vision problems are common, including deterioration. Patients cannot always report vision problems due to communication difficulties. Vision problems are often underdiagnosed. Affects communication, social skills, behaviour, and functioning. | Vision problems are not often reported by patients or their caregivers. |
| 1. **Relationships** | Relationships are an essential component of having a good quality of life. Patients may have difficulties establishing and maintaining relationships due to a lack of social skills. Affects mental health, well-being, and functioning. Lack of relationships can cause frustration. Support from a psychologist is needed to help them establish and maintain relationships. Gives insight into the level of functioning/ID. Difficult PRO to discuss with patients. | Patients can be very social. Patients do not always want to discuss their relationships with their HCP. |
| 1. **Sensory under-responsivity** | Sometimes reported by patients. Can have an impact on well-being and functioning. Important to discuss with a HCP to provide appropriate advice and treatment. | Over-responsivity is more common. |
| 1. **Chewing and swallowing** | Patients can have trouble chewing due to low muscle tone or sensory processing difficulties. Can be life threatening. Some patients receive tube feeding because they cannot chew properly. Can affect well-being and functioning. Can lead to social exclusion. | Not relevant to all patients. |
| 1. **Respiratory symptoms** | Sleep apnoea is common; can affect sleep and behaviour. Can impact well-being and functioning, and sometimes even survival. | Respiratory symptoms are less common. Not all patients need a comprehensive respiratory assessment. |
| 1. **Sexual functioning** | Important to know patient’s sexual needs. Parents are not always aware of the sexual needs of their children. Inappropriate sexual behaviour can be an issue and needs to be addressed. Being physically impaired affects sexual functioning. Patients may have difficulties achieving orgasm. Often underreported but has a high impact on daily life. HCPs need to give education about this PRO; patients need to know the consequence and risks of having sexual intercourse. Very rarely discussed by HCPs. | Not relevant for children. Not all patients are interested in sex. Patients find it difficult to discuss this PRO with a HCP. |
| 1. **Depressive symptoms** | Depressive symptoms are common (sometimes as a side-effect of medication) among patients. Depressive symptoms can have a significant impact on well-being, functioning, and quality of life. It is an important PRO to discuss with the HCP (especially since patients do not always report depressive symptoms and to prevent suicidal actions) and provide treatment. | Not everyone experiences depressive symptoms. Especially, patients with severe or profound intellectual disability may not experience depressive symptoms. It can be a heavy PRO to discuss. |
| 1. **Hearing** | Ear infections or hearing impairments are common. Patients cannot always report hearing problems due to communication difficulties. Hearing problems can affect communication, social skills, behaviour, and daily functioning. | Hearing problems are not often reported by patients or their caregivers. |
| 1. **Itch** | Can effect daily functioning. May arise after showering. May be a result of allergies or dry skin. | Not an issue for patients. Not often reported by patients or caregivers. Not necessary to discuss itch. |
